# Supplementary material for: Molecular flexibility of DNA as a key determinant of RAD51 recruitment
Source: EMBO J. 2020 Jan 14;39(7):e103002. doi: 10.15252/embj.2019103002 (PMC7110135; doi:10.15252/embj.2019103002)
Supplement: Supplementary file 2 — Expanded View Figures PDF [file EMBJ-39-e103002-s002.pdf]

Expanded View Figures

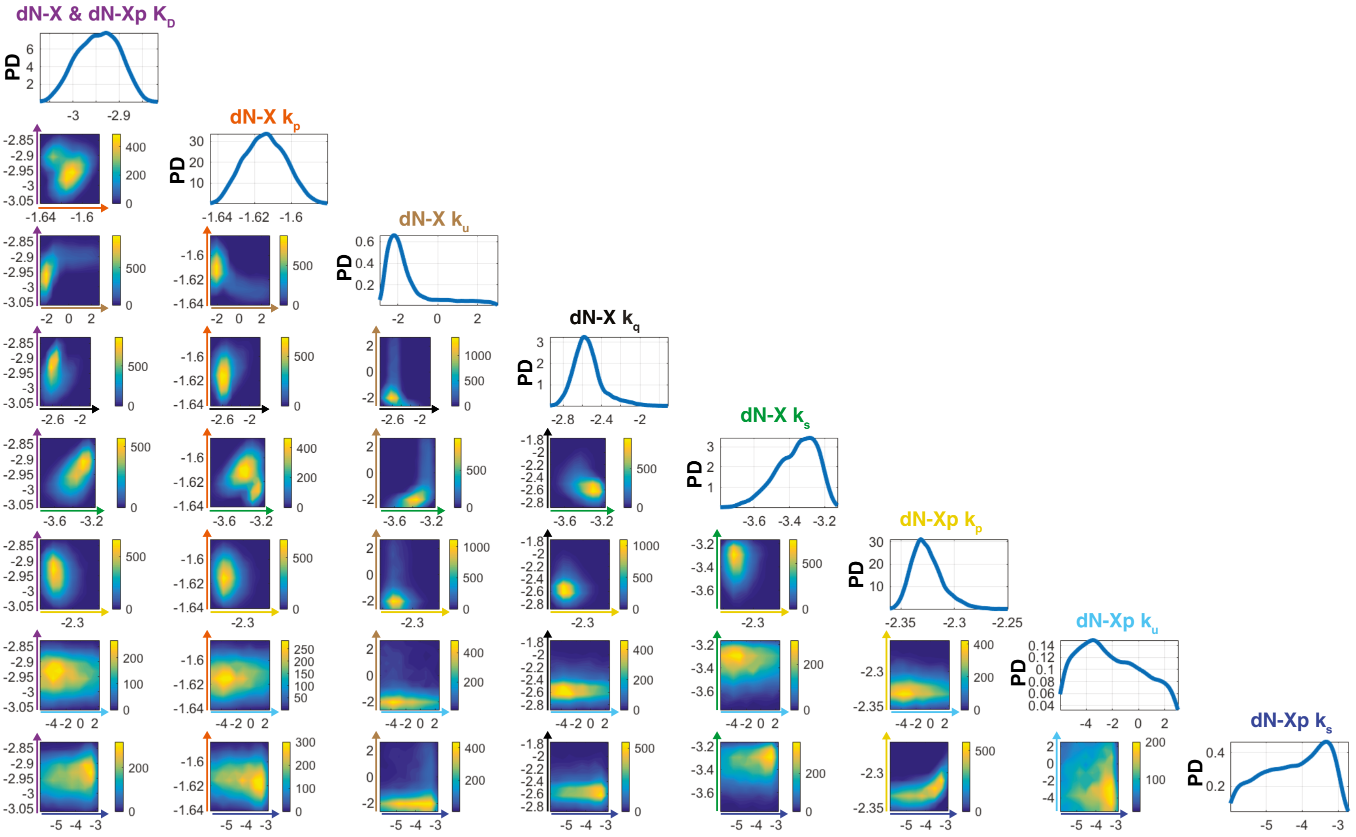

Figure EV1. ABC-SMC for WT RAD51 SPR data fitting.

ABC-SMC probability densities for the simultaneous WT RAD51 ssDNA (Fig 3A) and dsDNA model (Fig 3B) fits of the ssDNA dN-X (Fig 2B) and dsDNA dN-Xp (Fig 2D) SPR data. Heat maps indicate particle frequencies and describe any pairwise correlations between model parameters (dN-X & dN-Xp K_D, dN-X k_p, k_u, k_q, k_s, dN-Xp k_p, k_u, k_s). All heat map axes are in log–log scale and are labelled according to the corresponding parameter colour: dN-X & dN-Xp K_D (purple), dN-X k_p (orange), dN-X k_u (brown), dN-X k_q (black), dN-X k_s (green), dN-Xp k_p (yellow), dN-Xp k_u (cyan) and dN-Xp k_s (blue). A high particle frequency corresponds to a good model fit to the data for the specified parameter pair. The probability densities for individual parameters are presented along the top left diagonal and were estimated using a Gaussian kernel. Particles were initialised via log-uniform priors with the following lower and upper bounds: 10^{-4} < dN-X & dN-Xp K_D < 10^{-1}, 10^{-4} < dN-X k_p < 10^{4}, 10^{-6} < dN-X k_u < 10^{3}, 10^{-6} < dN-X k_q < 10^{3}, 10^{-6} < dN-X k_s < 10^{3}, 10^{-4} < dN-Xp k_p < 10^{4}, 10^{-6} < dN-Xp k_u < 10^{3} and 10^{-6} < dN-Xp k_s < 10^{3}. PD, probability density.

Source data are available online for this figure.

© 2020 The Authors

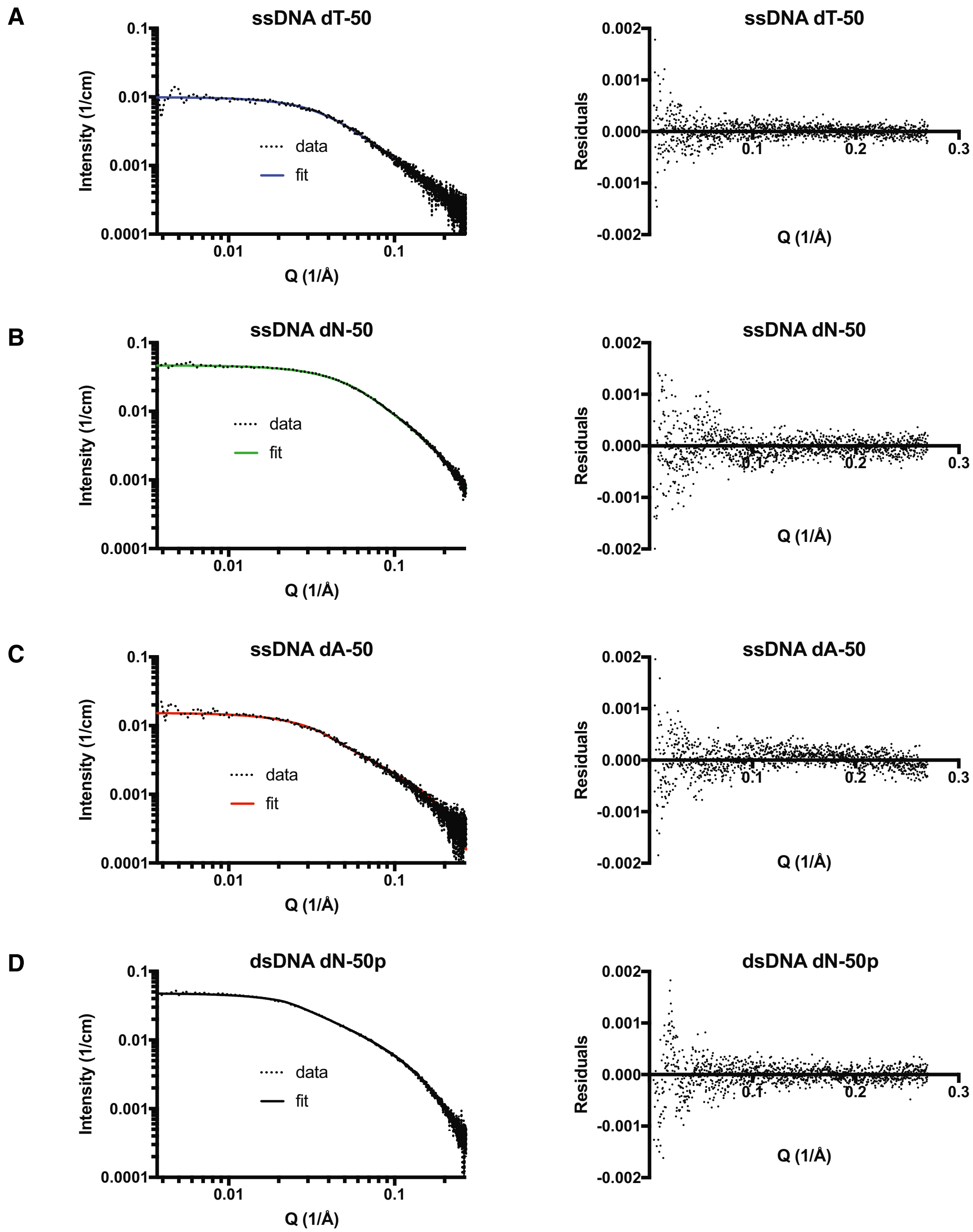

Figure EV2. The flexibility measurements of DNA oligonucleotides used in this study.

A A depiction of the Bend-to-Capture (BTC) mechanism. RAD51 generates two sequential, non-covalent interactions (a RAD51 protomer–protomer interaction and a RAD51-DNA interaction, or vice versa) faster on flexible DNA (depicted as ssDNA) compared to rigid DNA (depicted as dsDNA). The free RAD51 monomer or polymer in solution (here depicted as a monomer) to be incorporated into the growing polymer is shown with an asterisk.

B–D The flexibilities of ssDNA dT-50 (A), dN-50 (B), dA-50 (C) and dsDNA dN-50p (D) were assessed by small-angle X-ray scattering (SAXS). The left panels show the absolute scattering intensity (cm^{-1}) measured as a function of the scattering vector ($Å^{-1}$). The flexible cylinder model was fit to the data (fitting range: 0.0037–0.27 $Å^{-1}$) to predict the kuhn lengths (persistence length = kuhn length/2). The right panels show residuals of the flexible cylinder model fits.

Source data are available online for this figure.

© 2020 The Authors The EMBO Journal e103002 | 2020 17 of 15

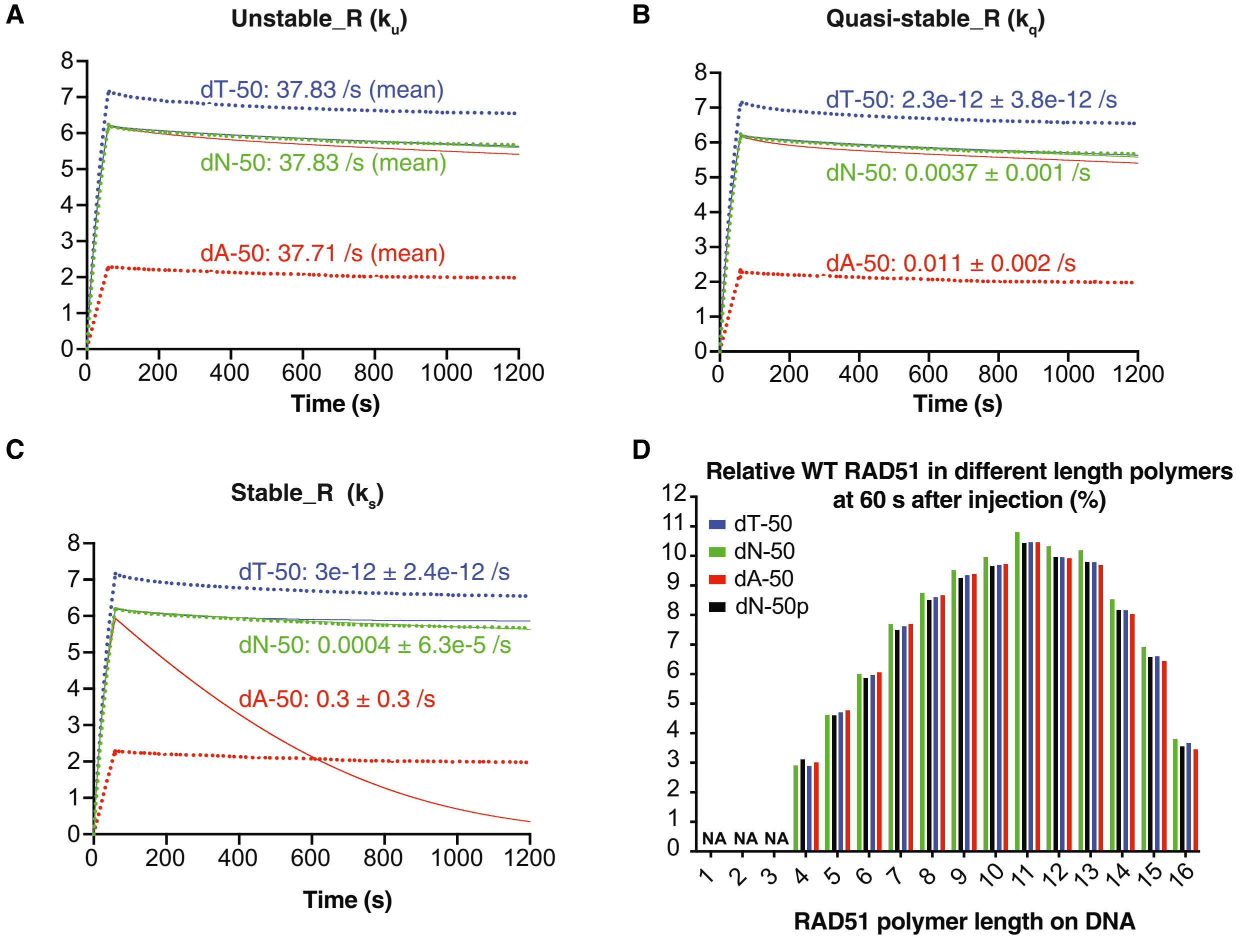

Figure EV3. Evaluation of WT RAD51 ssDNA model for ssDNA of variable flexibility.

A–C The mean number of WT RAD51 bound to respective DNA oligonucleotides is plotted versus time, as detected by SPR (dotted lines). The curves for the ssDNA dT-50, dN-50 and dA-50 were then fitted using lsqcurvefit (MATLAB) by varying a single parameter. The ODE models varying the unstable reverse rate constant (k_u) (A), the quasi-stable reverse rate constant (k_q) (B) and the stable reverse rate constant (k_s) (C) are shown. Varying either k_u, k_q or k_s does not generate accurate model fits. dN-X k_u was undetermined (Fig 3A), and for this reason, fit values in A are reported only as means.

D Model prediction of the % WT RAD51 in different length polymers on ssDNA dN-50, dT-50, dA-50 and dsDNA dN-50p after a 60-s injection of WT RAD51 using models from Fig 3A and B. Parameter values in the ssDNA model were set to the mean values as identified in Fig 3A, and % WT RAD51 values were calculated by multiplying the polymer concentrations by their respective polymer length and dividing each value by the total amount of RAD51 bound to DNA after a 60-s injection (i.e. % WT RAD51 = [*n*-mer] * *n*/[WT $RAD51_{total\ DNA\text{-}bound}$]). dN-X and dN-Xp k_u were undetermined (Fig 3A and B), and for this reason, the values for % WT RAD51 in monomers, dimers and trimers were not reported and labelled as NA (not applicable).

Source data are available online for this figure.

 © 2020 The Authors

A

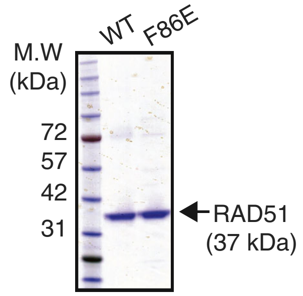

B

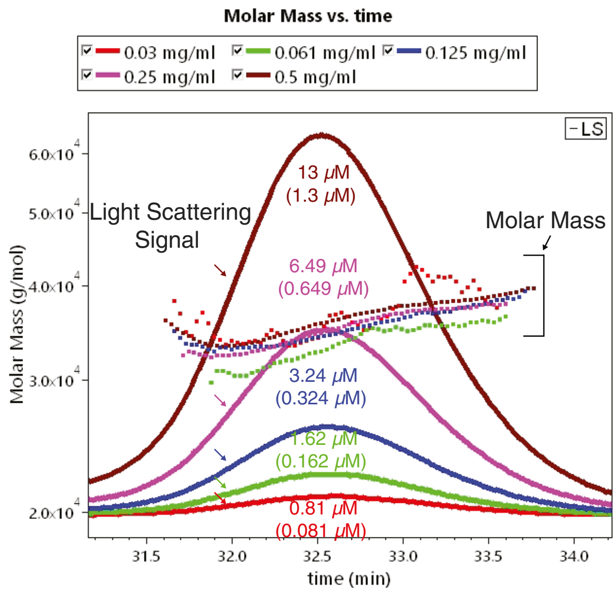

C

[F86E] (mg/ml)	Mw (kDa)	RSE
0.03	36.2	4.6%
0.061	33.3	1.1%
0.125	34.9	0.8%
0.25	34.9	0.2%
0.5	35.9	0.4%
Average	35	

D

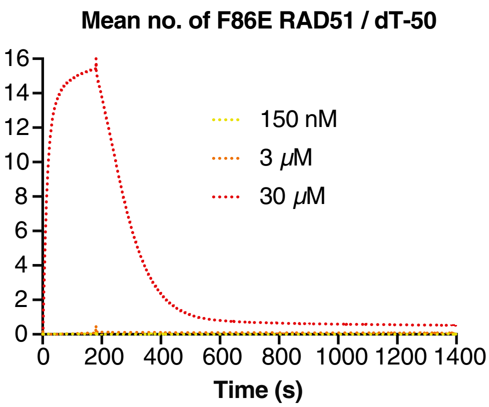

E

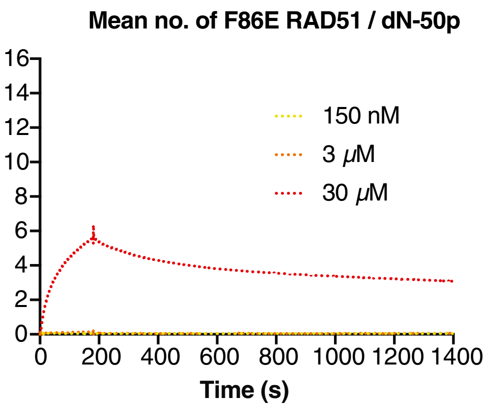

Figure EV4. Biochemical characterisation of F86E RAD51 used in this study.

A Purified WT and F86E RAD51 on an SDS–PAGE gel are visualised by Coomassie blue staining.

B Size-exclusion multi-angle light scattering (SEC-MALS) curves for F86E RAD51 at five injection concentrations as indicated. The Superdex200 10/300 GL size-exclusion column leads to a ~ 10-fold dilution of the injected sample. Hence, approximate elution concentrations are given in brackets below the injection concentrations. Dotted lines represent the molar mass derived values for each elution peak, and solid lines represent light scattering. F86E RAD51 concentrations are shown in both mg/ml and μM.

C Calculated molecular weights of the five F86E RAD51 elution peaks. Data were analysed using the ASTRA 6.1 chromatography software for SEC-MALS. RSE: relative standard error.

D, E F86E RAD51 displays reduced affinity for ssDNA and dsDNA. The mean number of F86E RAD51 bound to the ssDNA dT-50 (D) and the dsDNA dN-50p (E) at the colour-coded concentrations is plotted versus time, as detected by SPR.

Source data are available online for this figure.

© 2020 The Authors

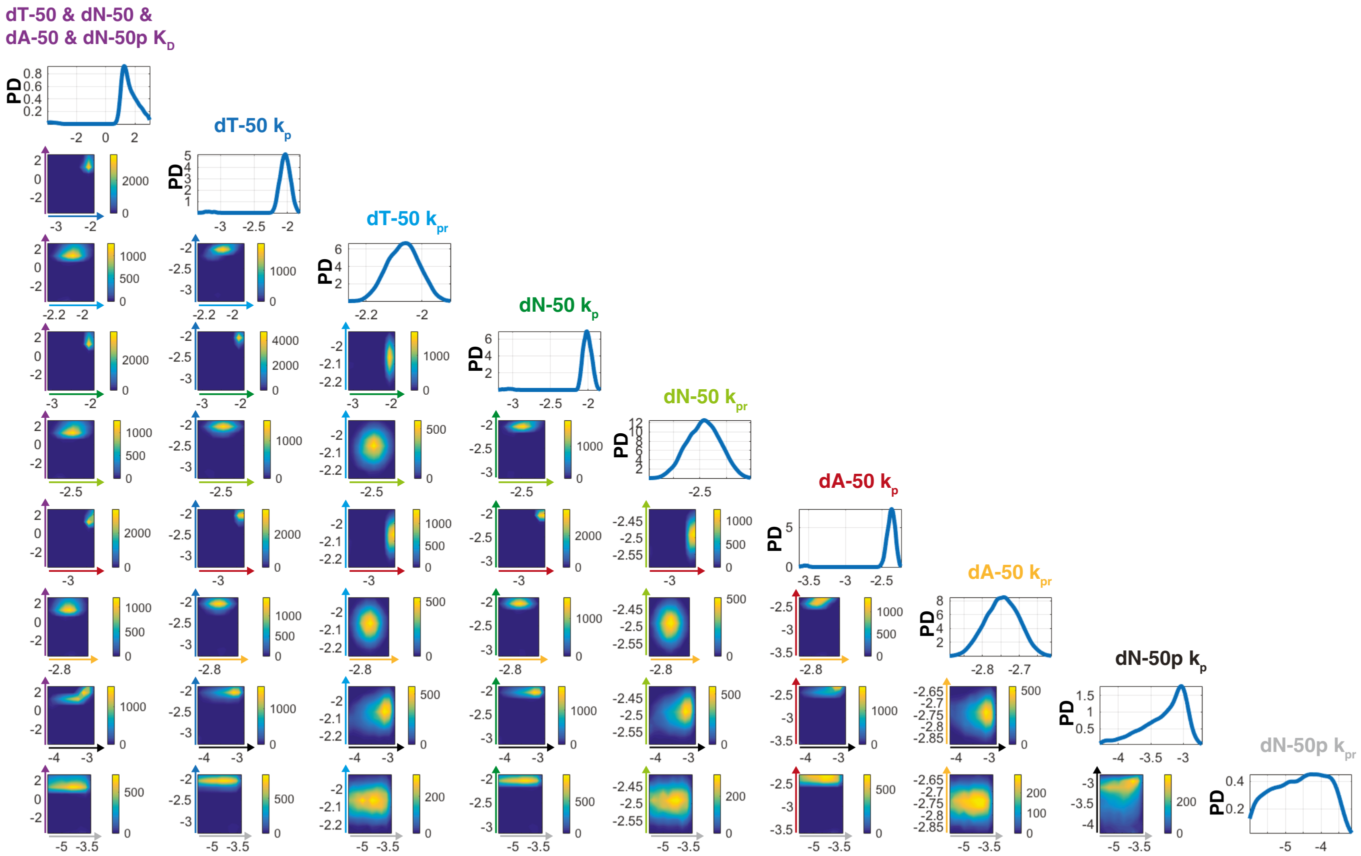

Figure EV5. ABC-SMC for F86E RAD51 kinetic data fitting.

ABC-SMC probability densities for the F86E RAD51 ssDNA/dsDNA model (Fig 5D) fit of the ssDNA dN-50, dA-50, dT-50 and dsDNA dN-50p SPR data. All four SPR curves were fit simultaneously using the same model. k_p and k_{pr} values were individually fit to each curve. A single K_D was fit to all four curves. Heat maps indicate particle frequencies and describe any pairwise correlations between model parameters (dT-50 & dN-50 & dA-50 & dN-50p K_D, dT-50 k_p, k_{pr}, dN-50 k_p, k_{pr}, dA-50 k_p, k_{pr}, dN-50p k_p, k_{pr}). All heat map axes are in log–log scale and are labelled according to the corresponding parameter colour: dN-X & dN-Xp K_D (purple), dT-50 k_p (blue), dT-50 k_{pr} (cyan), dN-50 k_p (green), dN-50 k_{pr} (light green), dA-50 k_p (red), dA-50 k_{pr} (salmon), dN-50p k_p (black) and dN-50p k_{pr} (grey). A high particle frequency corresponds to a good model fit to the data for the specified parameter pair. The probability densities for individual parameters are presented along the top left diagonal and were estimated using a Gaussian kernel. Particles were initialised via log-uniform priors with the following lower and upper bounds: $10^{-4} < K_D < 10^3$, $10^{-6} <$ dT-50 $k_p < 10^4$, $10^{-6} <$ dT-50 $k_{pr} < 10^3$, $10^{-6} <$ dN-50 $k_p < 10^4$, $10^{-6} <$ dN-50 $k_{pr} < 10^3$, $10^{-6} <$ dA-50 $k_p < 10^4$, $10^{-6} <$ dA-50 $k_{pr} < 10^3$, $10^{-6} <$ dN-50p $k_p < 10^4$ and $10^{-6} <$ dN-50p $k_{pr} < 10^3$. PD, probability density.

Source data are available online for this figure.

© 2020 The Authors
